# A transferable heterogeneous two-hybrid system in *Escherichia coli *based on polyhydroxyalkanoates synthesis regulatory protein PhaR

**DOI:** 10.1186/1475-2859-10-21

**Published:** 2011-04-09

**Authors:** Zhi-Hui Wang, Ping Ma, Jiong Chen, Jing Zhang, Chong-Bo Chen, Guo-Qiang Chen

**Affiliations:** 1 Multidisciplinary Research Center, Shantou University, Shantou 515063, Guangdong, China; 2 Department of Biological Science and Biotechnology, School of Life Sciences, Tsinghua University, Beijing 100084, China

## Abstract

**Background:**

Polyhydroxyalkanoate (PHA) synthesis regulatory protein PhaR contains a DNA binding domain (DBD) and a PHA granule binding domain (GBD), it anchors to the promoter region of PHA granule-associated protein (PhaP) to repress *phaP *expression. However, PhaR will bind to PHB granules and be released from *phaP *promoter region when PHA granules are formed *in vivo*, initiating expression of *phaP *gene. Based on this regulatory mechanism, a bacterial two-hybrid system was developed: PhaR was separated into two parts: DBD was used to fuse with the bait, GBD with the prey, and *phaP *was replaced by a reporter gene *lacZ*. However, GBD protein expressed *in vivo *formed inclusion bodies. Thus, PhaP with strong binding ability to PHB granules was employed to replace GBD.

**Results:**

Three model interaction partners bFos, bJun and bATF2 were used to study the feasibility of this bacterial two-hybrid system compared with the controls lacking one or more essential elements of this system. Results showed that bFos, bJun and bATF2 bound tightly in pairs to allow strong expression of β-galactosidase in different expression levels. In contrast, very weak β-galactosidase activity was detected in all control groups.

**Conclusion:**

β-Galactosidase activity level precisely correlated with the interaction force of tested protein pairs, and very weak β-galactosidase expression was detected throughout the control groups, which demonstrated the feasibility of this system for studying protein interactions.

## Introduction

Protein-protein interactions (PPIs) are essential in virtually all biological processes [1]. In the past two decades, a number of technologies to identify interacting proteins or to study these interactions have been extensively developed [2-6]. Among them, the most widely and successfully used methodology is the yeast two-hybrid system, originally developed by Chien et al [3], it exploits hybrid genes to detect protein-protein interactions by means of expression activation of a reporter gene [4]. Recently, a number of bacterial-based hybrid systems have been studied and become widely used. So far, studies of protein interactions in bacteria have centered on fusions to transcriptional repressors such as λcI, LexA or AraC, transcriptional activators involving the recruitment of RNA polymerase or the dimerization of the *Vibrio cholera *ToxR, complementation of biosynthetic enzymes such as dihydrofolate reductase, or signaling enzymes, e.g., the *Bordetella pertussis *adenylate cyclase [5]. By contrast, bacteria-based systems present advantages over yeast-based technologies, such as lack of cellular compartmentalization, faster growth and higher transformation efficiencies that are attainable permitting rapid and more efficient screening of complex libraries [6]. In spite of these advantages, all bacterial strategies have their drawbacks, including the need to employ the host intrinsic proteins for strategies of enzyme complementation, which will result in possible false positive outcomes, so host self-existed enzymes should be deleted from the host genome to eliminate interferences. This increases the complexity of genetic manipulation, and such technology developed could not be transferred to other bacterial strains. Therefore, a simple and transferable hybrid strategy should be developed to meet rising experimental demands.

**Figure 1:**
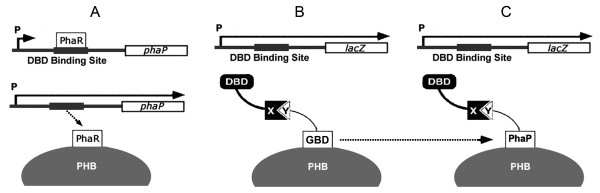
**The principle of PhaR regulation and its application in two-hybrid system**. (A) PhaR can specifically bind to the *phaP *promoter to repress its expression, however, when PHB granules are produced, PhaR will prefer to bind PHB and release from the *phaP *promoter, liberating PhaP expression. (B) PhaR has two separate domains, a DNA binding domain (DBD) and a PHB granules binding domain (GBD), each domain fuses with a protein (X and Y), if × and Y have an interaction, this power will direct to a complex DBD-X:Y-GBD, just like a reconstituted "PhaR", so when PHB granules exist, this reconstituted "PhaR" will drop from DBD binding site and release the expression of LacZ reporter. (C) GBD and its fusions expressed *in vivo *were insoluble, so PhaP also with a strong PHB binding ability was employed to replace GBD in this study.

Polyhydroxyalkanoates (PHA) are biodegradable polyesters produced as intracellular carbon and energy storage materials by a wide variety of bacteria [7,8] and genetically engineered *Saccharomyces cerevisiae *[9]. Polyhydroxybutyrate (PHB) is an important member of PHA family, its synthesis is regulated by several proteins including PHB synthase (PhaC), granule-associated protein PhaP (also called phasin) and regulatory protein (PhaR) [10]. In model PHA producing strain *Ralstonia eutropha *H16, PhaR functions as a repressor or autoregulator for the expression of PhaP and PhaR itself, both of which can tightly bind to PHB granules [11]. PhaR contains a DNA binding domain (DBD) and a PHB granule binding domain (GBD) and binds to the *phaP *promoter region to repress its expression. However, when PHB granules are produced *in vivo*, PhaR will bind to PHB granules and dissociate from the *phaP *promoter region, allowing the expression of *phaP *[12] (Figure 1A).

Fos, Jun and ATF2 are transcription factors with basic leucine zipper (bZIP) domain. The relative dimerization efficiencies among the bZIP domains of the Fos (bFos), Jun (bJun) and ATF2 (bATF2) have been determined by the multicolour bimolecular fluorescence complementation (BiFC) assay [13]. Apparently, the bZIP domains of Fos, Jun and ATF2 can interact with each other in all pairwise combinations *in vitro *and *in vivo *[14]. In living cells, bJun:bFos heterodimers form more efficiently than either bFos:bATF2 or bJun:bATF2 heterodimers. Moreover, bJun:bFos heterodimers and bJun:bJun homodimers can coexist in cells with a content ratio of 60%:35% [15].

In this investigation, PhaR was intended to be developed into a platform for protein interaction study. To achieve this, PhaR was originally divided into two parts, namely, DBD and GBD. DBD fused to a bait protein, GBD fused to a prey protein, and *phaP *gene under the control of its native promoter was replaced by a reporter gene *lacZ *(Figure 1B). If the bait and the prey interact with each other, the new DBD-bait:prey-GBD complex will drop from the *phaP *promoter region, and LacZ will be expressed, with its expression level indicating the interaction strength of the bait and the prey. However, GBD protein expressed *in vivo *formed an inclusion body probably due to its high contents of hydrophobic amino acid residues. To avoid this, PhaP also having strong binding ability to PHB granule was employed to replace GBD (Figure 1C). Also in this study, three model interaction partners, bFos, bJun and bATF2, were used to test the feasibility of this system. Pairwise interactions among these proteins leaded to different levels of LacZ expression, directly reflecting their factual interaction strengths [15]. Since all elements involved including PHB synthesis operon *phaCAB*, DBD, *phaP *and *phaP *promoter are extrinsic in *E. coli*, false positive outcomes could be avoided. Moreover, the plasmids harboring these elements can be transferred to other appropriate host organisms for specific purpose, such as post-translational modification and glycosylation, allowing the heterogeneous two-hybrid system to become more extendable.

## Materials and methods

### Conserved domain prediction of PhaR

The conserved domain prediction of PhaR (GenBank: YP_725943) was conducted using Pfam software http://pfam.sanger.ac.uk/search/sequence. Results showed that the DNA-binding domain was in the N-terminal region (10^th ^to 73^th^). To retain full DNA-binding ability, the 1^st ^to 106^th ^amino acid segment of PhaR as DBD was chosen for this study [12].

### Bacterial strains and plasmids

The bacterial strains and plasmids used in this study were listed in Table 1. Primers used to construct relevant plasmids were listed in Additional file 1, Table S1. The flow charts of reconstruction of these plasmids are described in details in complementary data (Additional file 1, Figs. S1-S6). *E. coli *XL1-Blue as the host strain was used to transform all plasmids, to conduct protein expression and PHB production. *Ralstonia eutropha *H16 was the source of *phaP *gene with its native promoter (Genbank: AF079155) and DBD encoding sequence. Plasmid pBHR68 [16] harboring PHB synthesis operon *phaCAB *was modified to generate plasmids for expressing PhaP-prey and for production of PHB granules. Simultaneously, plasmid pACYC184 (Fermentas, MBI), compatible with pBHR68, was employed to express DBD-bait and the reporter gene (*lacZ*) cloned from plasmid pPI-LacZ [17]. bFos (CGGLTDTLQAETDQLEDKKSALQTEIANLLKEKEKLEFILAAY), bJun (CGGRIARLEEKVKTLKAQNSELASTANMLREQVAQLKQKVMNY) [18] and bATF2 (GRRRRAANEDPDEKRRKFLERNRAAASRCRQKRKVWVQSLEKKAEDLSSLNGQLQSEVTLLRNEVAQLKQLLLAH)[19] were used as model bait and prey proteins, respectively. The gene segments of *bJun*, *bFos *and *bATF2 *were synthesized by Invitrogen (Guangzhou, China). All plasmids were confirmed by gene sequencing by Invitrogen (Guangzhou, China).

**Table 1:** Bacterial strains and plasmids used in this study

Strains & plasmids	Description	Source or Reference
Strains		
*E. coli *XL1-Blue	*endA1 gyrA96 *(*nalR*) *thi-1 recA1 relA1 lac glnV44 *F'[::Tn10 *proAB+ lacIq *Δ(*lacZ*) M15] *hsdR17 *(r^K- ^m^K+^)	Stratagene
*R. eutropha *H16	Wild type (PHB^+^)	DSMZ 428
Plasmids		
pBHR68	Amp^r^, pBluescript SK^-^, harboring PHB synthesis operon from *R. eutropha *(*phaCAB *operon), used to produce PHB granules	16
pP-CAB	Amp^r ^, pBHR68:*phaP *under control of *phaCAB *promoter	This study
pFos-P-CAB	Amp^r ^, pBHR68: *bFos-phaP *under control of *phaCAB *promoter	This study
pJun-P-CAB	Amp^r ^, pBHR68: *bJun-phaP *under control of *phaCAB *promoter	This study
pATF2-P-CAB	Amp^r ^, pBHR68: *bATF2-phaP *under control of *phaCAB *promoter	This study
pFos	Amp^r ^, pBHR68Δ(*phaCAB*):*bFos *under control of *phaCAB *promoter	This study
pFos-P	Amp^r ^, pBHR68Δ(*phaCAB*): *bFos-phaP *under control of *phaCAB *promoter	This study
pACYC184	Cm^r^, Tet^r^, cloning vector	Fermentas
pOZ	Cm^r^, pACYC184:*lacZ *under control of *phaP *promoter	This study
pDBD-Jun-Z	Cm^r^, pOZ:*DBD-bJun *under control of *phaCAB *promoter	This study
pDBD-Z	Cm^r^, pOZ:*DBD *under control of *phaCAB *promoter	This study
pPI-LacZ	Amp^r^, pTWIN2:*phaP-intein-lacZ*	17

### Plasmid designs

Several genes were involved in this bacterial two-hybrid system including DBD sequence, *phaP*, *lacZ *under *phaP *promoter and PHB synthesis operon *phaCAB*. In this study, two compatible vectors, pACYC184 and pBHR68 were chosen to harbor related genes. DBD sequence and *lacZ *under *phaP *promoter were cloned to pACYC184, a low copy number vector (Additional file 1, Fig. S6). PhaP gene and PHB synthesis operon *phaCAB *were cloned to pBHR68, a high copy number vector (Additional file 1, Fig. S2). This aimed to achieve an excessive expression of PhaP (much more than DBD), so that all DBD-bait fusions had full access to prey-PhaP fusion. Simultaneously, a high copy number of PHB synthesis operon may help to produce more PHB granules. Sufficient PHB granules should ensure adequate space for attachment of all expressed PhaP or its fusion proteins. This is supported by previous study [20], after the PHB granules were removed from crude cell extracts via mild centrifugation, no PhaP was detected in culture supernatants using Western Blotting, indicating that all PhaP expressed *in vivo *were attached on PHB granules. These results pointed to an ideal situation: as long as the interaction of bait and prey is strong enough, all DBD-bait proteins can be caught by prey-PhaP to form a DBD-bait:prey-PhaP complex, which became attached to PHB granules, leading to the release of DNA repression on LacZ expression. Therefore, the expression level of LacZ depends on the interaction strengths of bait and prey.

### Cultivation of strains, protein expression and PHB production

For the purpose of gene clone, recombinants of *E. coli *XL1-Blue were grown at 37°C and 200 rpm (FUMA QYC2112, Shanghai, China) in Luria-Bertani medium containing 1% w/v Bacto tryptone, 0.5% w/v yeast extract and 1% w/v NaCl overnight. When needed, tetracycline (50 μg/ml) or ampicillin (100 μg/ml) or chloramphenicol (34 μg/ml) was added to the medium. For the sake of protein expression and PHB granules production, the recombinants of *E. coli *XL1-Blue were cultivated at 37°C and 200 rpm (FUMA QYC2112, Shanghai, China) for 14 h in Luria-Bertani medium supplemented with 20 g/L glucose, to ensure sufficient PHB granules accumulation for this study as was also reported [21].

### Assays of β-galactosidase activity

β-Galactosidase was employed as a reporter due to its ease for quantitative analysis. β-Galactosidase assays were performed using Bacterial X-Gal Staining Kit (GENMED, Shanghai, China). The OD values were quantitatively determined at 420 nm with a microplate reader (Beckman Coulter DU800, USA). OD values were applied to the following formula:

Where, according to the manufacturer's protocol, "1.7" represents total reaction solution volume (ml), "0.1" sample volume (ml), "0.0045" is molar absorption coefficient of *o*-Nitrophenol at 420 nm (ml/nmol/cm), "T" stands for the reaction time (min): the reaction is defined as the time consumed started from placing the reaction solution into 37°C thermostatic water-bath to the change of solution color to light yellow, "1" reflects the light path length (cm), "C" the protein concentrations of samples (mg/ml). Each value represents the average of three parallel samples.

### Statistical Analysis

The statistical significance was evaluated by Duncan multiple range test [22], which was used to perform analysis of significant differences for β-galactosidase activity data. Probability values of *p *< 0.01 were interpreted as denoting statistical significance. Statistical analyses were performed by PRISM software (GraphPad, San Diego).

## Results

### Identification of DNA binding domain and PHB granule binding domain of PhaR

**Figure 2:**
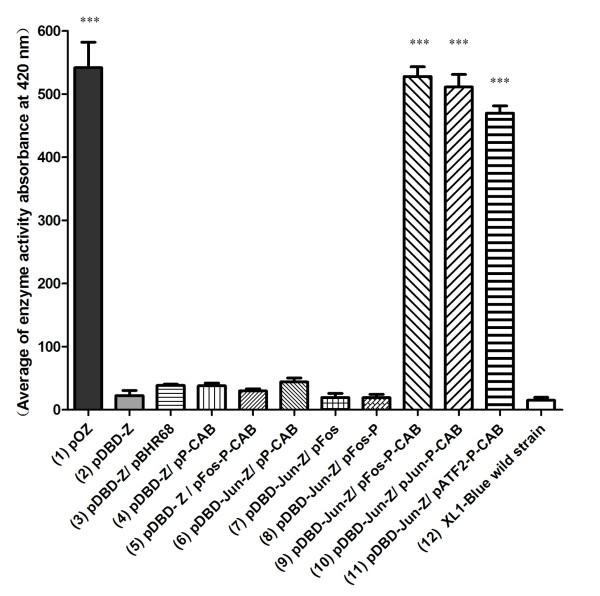
**β-Galactosidase activity assay**. *E. coli *XL1-Blue harboring relative plasmids were cultured in LB medium with 20 g/L glucose for 14 h at 37°C, 200 rpm, then β-galactosidase activity was assayed by Bacterial X-Gal Staining Kit (GENMED, Shanghai, China). Bar 1: LacZ has a strong expression under control of *phaP *promoter (positive control); Bar 2: LacZ expression can be repressed by DBD; Bar 3-5: PHB granules, PhaP, and bFos-PhaP can not pull down DBD from *phaP *promoter to release LacZ expression; Bar 6: DBD-bJun has no interaction with PhaP; Bar 7,8: When PHB granules are absent, the interaction of DBD-bJun with bFos and bFos-PhaP still can not liberate LacZ expression, indicating PHB granules are essential; Bar9-11: When PHB are present, interactions between bJun:bFos, bJun:bJun and bJun:bATF2 can liberate LacZ expression; Bar 12: LacZ activity detected in *E. coli *XL1-Blue wild strain (negative control). Each bar represents the mean value ± standard deviation. Three asterisks *** (p < 0.001) denotes significant differences between mean values measured in other strains compared with pDBD-Z transformed strain. The original data for Duncan multiple range tests were listed in Table S2 (see additional files).

**Figure 3:**
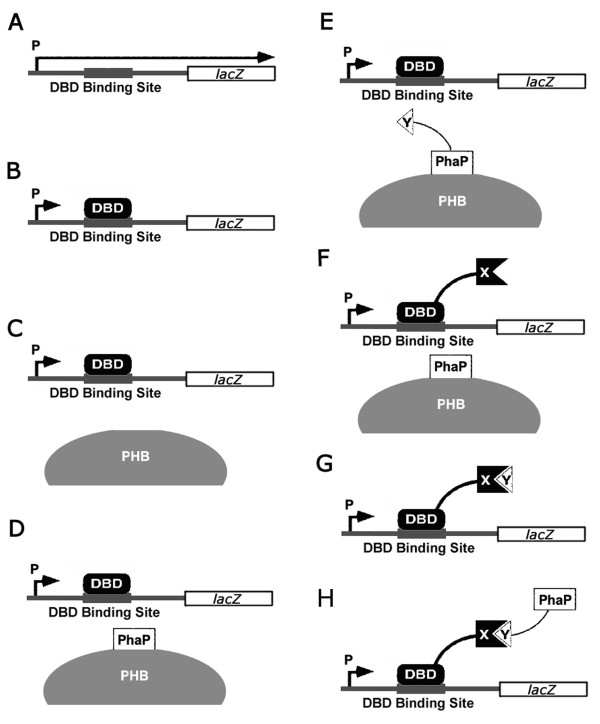
**Experiment diagram of interaction between essential elements in this two-hybrid system**. In order to examine whether interactions happen among several essential elements involved in this two-hybrid system, such as *phaP *promoter, DBD, PhaP, *lacZ *reporter gene, interaction protein partners and PHB granules. *E. coli *XL1-Blue harboring plasmids were cultured to detect the possible interaction between these elements by detecting β-galactosidase activity. DBD regulates LacZ expression by turning DBD on/off the DBD binding site of *phaP *promoter. (A) pOZ, the promoter of *phaP *can give rise to strong expression of LacZ; (B) pDBD-Z, DBD can suppress LacZ expression by binding on DBD binding site of *phaP *promoter; (C) pDBD-Z and pBHR68, PHB granules can not pull down DBD from DBD binding site, indicating DBD has no interaction with PHB granules; (D) pDBD-Z and pP-CAB, similar with (C), PhaP can not pull down DBD, indicating DBD has no interaction with PhaP; (E) pDBD-Z and pFos-P-CAB, similarly, DBD has no interaction with PhaP-bFos fusion; (F) pDBD-Jun-Z and pP-CAB, similarly, DBD-bJun has no interacton with PhaP; (G) pDBD-Jun-Z and pFos, when PHB granules are absent, the interaction of DBD-bJun and bFos can not give rise to LacZ expression; (H) pDBD-Jun-Z and pFos-P, when PHB granules are absent, the interaction of DBD-bJun and PhaP-bFos can not give rise to LacZ expression; These assays were used to eliminate the influences of unspecific interaction, which was so called background noise. X: bait protein; Y: prey protein.

PhaR from *Paracoccus denitrificans *(PhaR_Pd_) with two separate domains that bind respectively to target DNA and PHB granules was investigated by deletion mutation and gel shift assay [12]. A tertiary structure prediction of PhaR_Pd _with Pfam software was performed. The N-terminal 10^th ^to 73^th ^amino acid motif was predicted to be the DNA binding domain. However, PhaR_Pd _with a C-terminal deletion mutation from 73^th ^to 195^th ^resulted in loss of DNA binding ability. In contrast, PhaR_Pd _with a C-terminal deletion mutation from 164^th ^to 195^th ^retained its DNA binding ability. This phenomenon indicated that a part of amino acid segment after 73^th ^also contributed to DNA binding. Unlike PhaR_Pd_, no report was found regarding DBD and GBD of PhaR from *Ralstonia eutropha *(PhaR_Re_). When aligning the amino acid sequences of PhaR_Pd _and PhaR_Re _using ClustalW2 software http://www.ebi.ac.uk/Tools/clustalw2/index.html, no significant homologous fragment was found. However, results of a tertiary structure prediction indicated that both PhaR have a N-terminal 10^th ^to 73^th ^DNA binding domain and a 75^th ^to 115^th ^PHB granules binding domain. Interestingly, PhaR_Re _has an additional PHB binding domain from 126^th ^to 166^th^. Though the boundary between DBD and GBD of PhaR_Re _is not clear, it is necessary to obtain a DBD without PHB granules binding activity and a GBD without DNA binding activity for our bacterial two-hybrid system. Since the DNA binding activity of PhaR_Pd _was eliminated with deletion from 73^th ^to 195^th ^of its amino acid motif which is just behind the predicted DNA binding motif, we chose a compromise site to extend the DBD region from 1^st ^to 106^th ^of its amino acid motif, the left segment was selected as GBD. Fortunately, DBD and its fusion protein could still anchor on promoter region of *phaP *to repress the expression of LacZ when PHB granules were formed (Figure 2, bar 3 and Figure 3C). Certainly, a minimal DBD including a complete DNA binding domain can be obtained by further deletion mutations and gel shift assays. However, GBD and its fusion proteins were insoluble when expressed in *E. coli *(Data not shown), probably due to its high hydrophobic amino acid residues. This result was similar with those of N-terminal deleted mutants of PhaR_Pd_, which were found to also be insoluble when expressed in *E. coli *[12], indicating that N-terminal region is necessary for PhaR folding. To overcome the insolubility of GBD and its fusion proteins, PhaP also having a strong binding ability to PHB granules was chosen to replace GBD in this study. As expected, the strong β-galactosidase activities indicated that PhaP functioned properly as its GBD counterpart did when protein interactions occurred (Figure 1C and 2, bar 9-11).

### Study of the two-hybrid system using leucine zipper proteins Jun and Fos

Two compatible plasmids pACYC184 and pBHR68 were modified to express and produce DBD-X and reporter LacZ, PhaP-Y and PHA granules, respectively. × and Y stand for a pair of tested interacting proteins (Figure 1C). To study the feasibility of this system, the leucine zipper domains of transcription factors Fos and Jun, which have been confirmed to have a strong interaction, were chosen as model interaction partners. The reporter gene *lacZ *was placed under the control of the *phaP *promoter. In principle, DBD-Jun fusion protein binds continuously to the *phaP *promoter region as soon as they are produced, repressing the expression of LacZ until PHB granules begin to form. The interaction of bJun and bFos will force DBD-bJun and bFos-PhaP to form a new complex, namely, DBD-bJun:bFos-PhaP, which is similar to a reconstituted 'PhaR' protein. As soon as PHB granules are formed, DBD-bJun:bFos-PhaP will be released from the *phaP *promoter region, resulting in LacZ expression. As expected, a strong expression of LacZ was observed (Figure 2, bar 9). This is the first evidence showing the feasibility of this two-hybrid system.

### Confirmation of the LacZ expression only resulted from interacting proteins

In this bacterial two-hybrid system, there are several indispensable elements: *phaP *promoter, DBD, PhaP, *lacZ *reporter gene, interaction protein partners and PHB granules. Possible influences of interactions among these elements themselves would lead to false positive results, so experiments must be performed to confirm that the LacZ expression only derives from the interaction between protein pairs.

Firstly, we investigated whether LacZ could express normally under the control of *phaP *promoter. For this purpose, plasmid pOZ was constructed (Figure 2, bar 1 and Figure 3A), its expression resulted in a high β-galactosidase activity comparable with bJun and bFos interaction group (Figure 2, bar 9), confirming the effectiveness of *phaP *promoter to regulate LacZ expression.

Secondly, plasmid pDBD-Z was constructed to investigate whether DBD can inhibit LacZ expression when PHB granules are not available (Figure 2, bar 2 and Figure 3B). Only little β-galactosidase activity was observed when pDBD-Z was co-expressed compared with strong β-galactosidase activity observed when pOZ was expressed alone. Therefore, DBD can be considered to suppress LacZ expression.

To further exclude the possibility of LacZ expression by elements other than interaction protein partners, following studies were performed: To investigate whether DBD could interact with PHB granules, PhaP or bFos, their corresponding plasmid combinations (Figure 2, bar 3-5 and Figure 3C-E,) were transformed into *E. coli *XL1-Blue to produce these elements. If interaction happened, a false positive LacZ activity could be detected. Similarly, we detected whether PhaP could interact with bJun (Figure 2, bar 6 and Figure 3F), and whether the interaction between DBD-bJun and bFos-PhaP or bFos (Figure 2, bar 7,8 and Figure 3G,H) could also direct to LacZ expression when PHB granules were absent, in other words, whether PHB granules are essential for LacZ expression. Obviously, LacZ activities produced by all above groups of recombinants (Figure 2, bar 3-8) were on the similar low level as that detected as a background level shown by the wild type *E. coli *XL1-Blue containing no plasmid (Figure 2, bar 12). These results clearly demonstrated that the LacZ activity resulted only from bait and prey interaction.

### Study of the two-hybrid system using different interaction protein pairs

To further prove the feasibility of this two-hybrid system, two more interacting protein pairs, namely, bJun:bJun and bJun:bATF2 were employed. Plasmids pJun-P-CAB and pATF2-P-CAB harboring genes of bJun-PhaP and bATF2-PhaP, respectively, were transformed together with compatible plasmid pDBD-Jun-Z into *E. coli *XL1-Blue strains for their LacZ activity studies. From Figure 2, bar 9-11, it became clear that interacting pairs of bJun:bFos, bJun:bJun and bJun:bATF2 all showed strong LacZ expression with different strength levels, the strength of interaction correlated well with the β-galactosidase activity: bJun:bFos>bJun:bJun>bJun:bATF2.

## Discussion

### Feasibility study of this bacterial two-hybrid system

As a successful two-hybrid system for studying protein-protein interactions, the expression level of the reporter gene should directly reflect the interaction strength of proteins. In addition, background expression of the reporter gene should be as low as possible, because the low background noise will improve the accuracy of the method to study proteins with low interaction strengths.

To test the feasibility of our bacterial two-hybrid system, we chose three extensively studied model interaction partners, namely, bJun, bFos and bATF2. Previous multicolour bimolecular fluorescence complementation (BiFC) assays [15] showed that heterodimer bJun:bFos and homodimer bJun:bJun were able to coexist in living cells, and bJun:bFos had a 60% content in the cells compared with only 35% content of bJun:bJun, indicating that the interaction strength of bJun with bFos was stronger than that of bJun:bJun, the bJun:bATF2 being the weakest. That is to say that the strength order among these three pairs should be bJun:bFos>bJun:bJun>bJun:bATF2. This reported order was consistent with the β-galactosidase activity order of the three interacting protein pairs studied using our two-hybrid system (Figure 2, bar 9-11).

In principle, the background expression of the reporter gene may result from the undesired weak interaction between essential elements involved in this two-hybrid system, such as DBD and *phaP *promoter region, DBD and PhaP, DBD and PHB granules, PhaP and bait, and so on. In this study, several experiments to detect LacZ expression resulted from these undesired interactions were conducted (Figure 2). Results clearly revealed that β-galactosidase activity maintained at a very low level when PHB granules were not formed in the cells, while weak β-galactosidase activity could be detected when PHB granules existed *in vivo *although its activity was very low compared with that produced by the bJun:bFos group. The following reasons may explain this low β-galactosidase activity: Firstly, DBD used in this study consists of a part of predicted PHB binding motif and the full DNA binding motif predicted by Pfam software to retain a strong DNA binding ability. This part of predicted PHB binding motif included in the DBD used here may still have a low binding ability to PHB granules, thus, a very weak LacZ expression was initiated when PHB granules were produced. Secondly, at the very beginning, genes of DBD and LacZ were transcribed and translated simultaneously in cytoplasm of *E. coli*, the earliest expressed DBD could turn to anchor on promoter region of *lacZ*, repressing its subsequent transcription. However, previously transcribed LacZ mRNA can be translated normally, this part of LacZ may contribute to the small amount of β-galactosidase activity.

### Merits and drawbacks

Bacteria based two-hybrid systems allow the rapid analysis due to their faster growth rate compared with that of yeast, greater permeability to small molecules, absence of a requirement for nuclear localization and the possibility of studying proteins that are toxic when expressed in yeast [23].

When compared with other bacteria based two-hybrid systems, our system has additional advantages: all essential elements including PHB synthesis operon *phaCAB*, DBD, PhaP and the phaP promoter region are extrinsic in *E. coli*, therefore, we need not perform complicated genome DNA manipulations in the host strain, and need not worry about the influence of intrinsic components employed by other bacterial two-hybrid system. This is helpful to avoid intrinsic component derived false positive results. In addition, all essential elements used in our system are harbored by two compatible plasmids. Thus, this two-hybrid system can be easily transferred to other prokaryotic strains to obtain a better environment for production of bioactive proteins.

Results presented here have proven the feasibility of this bacterial two-hybrid system. However, further research should be carried out to obtain an optimal DBD without any PHB binding ability, which will contribute to a much lower background expression of reporter gene. On the other hand, PHB granules are essential for this two-hybrid system, even though plasmids can be modified for expression in eukaryotic strains such as yeast, the low yield of PHB in eukaryotic strains still limits the application of this system to study proteins requiring post-translational modification. PHB production ability is different depending on strains, experiments should be conducted to study proper culture conditions for sufficient PHB granules accumulation when this system is to be transferred to another strains. Sufficient PHB granules accumulation means sufficient space to attach all expressed PhaP fusion. For example, when bJun and bFos were employed as an interaction pair for our study, a relationship between β-galactosidase activity and culture time was established, the time point when β-galactosidase activity began entering its stationary phase was considered as the proper culture time (data not shown).

## Conclusion

A bacterial two-hybrid system based on two separate domains of PHA synthesis regulatory protein PhaR_Re _was established. DNA binding domain of PhaR_Re _fused with a bait protein was able to anchor on promoter region of *lacZ *reporter gene to repress its expression, PHB binding domain fused with a prey protein was found attached to PHB granules produced *in vivo*. The interaction of bait and prey forced two fusions to form a reconstituted "PhaR" which was dropped from the promoter region, releasing the expression of LacZ. LacZ expression level depended on strengths of bait and prey interaction. Results from three extensively studied model interaction proteins bJun, bFos and bATF2 confirmed the feasibility of our two-hybrid system.

## Competing interests

The authors declare that they have no competing interests.

## Authors' contributions

ZHW designed the study, performed some of the experiments and drafted the manuscript, PM, JC, JZ and CBC performed the experiments, GQC supervised the study and revised the manuscript. All authors read and approved the final manuscript.

## Supplementary Material

Additional file 1 **supplementary material - Table S1, Figs S1-S6**. Click here for file
