# Supplementary material for: A transferable heterogeneous two-hybrid system in Escherichia coli based on polyhydroxyalkanoates synthesis regulatory protein PhaR
Source: Microb Cell Fact. 2011 Apr 9;10:21. doi: 10.1186/1475-2859-10-21 (PMC3079617; doi:10.1186/1475-2859-10-21)
Supplement: Additional file 1 — supplementary material - Table S1, Figs S1-S6. [file 1475-2859-10-21-S1.DOC]

**Table S1.** Plasmids and primers used to clone DNA fragments from plasmids

| Plasmids | Primers |
| --- | --- |
| pBHR68P | P1: 5’-GGAATTCCATATGGATTTGATTGTCTCTCTGCCGTCACT-3’ |
|  | P2: 5’-GAAGATCTTGACCTGCCGGCCTGGTT-3’ |
|  | P3: 5’-ATACATATGGACGTCATGATCCTCACCCCGGAACA-3’ |
|  | P4: 5’-GGAAGATCTTCAGGCAGCCGTCGTCTTC-3’ |
| pFos-P-CAB | P5: 5’-CCCGGGCAAGTACCTTGCC-3’ |
|  | P6: 5’-TGCTCTAGATCGCCCCCGC-3’ |
| pP-CAB | P7: 5’-ATGGATTTGATTGTCTCTCTGCCGT-3’ |
|  | P8: 5’-ATGATCCTCACCCCGGAACAA-3’ |
| pFos | P9: 5’-GGAATTCCATATGGAAGAGCCCTCGA-3’ |
|  | P10: 5’-GAAGATCTCATATGTGCGGTGGTCTGAC-3’ |
|  | P11: 5’-GGAATTCCATATGGATTTGATTGTCTCTCTGCCGTCACT-3’ |
|  | P12: 5’-GAAGATCTTGACCTGCCGGCCTGGTT-3’ |
| pDBD | P13: 5’-GGAATTCCATATGGCCACGACCAAAAAA-3’ |
|  | P14: 5’-GAAGATCTACCGGTTTACTTCTTGTCCGGCTGGT-3’ |
|  | P15:5’-GGAATTCCATATGGATTTGATTGTCTCTCTGCCGTCACT-3’ |
|  | P16: 5’-GAAGATCTTGACCTGCCGGCCTGGTT-3’ |
| pACYC184-OP | P17: 5’-CCATCGATGTGCACATCCAGGTCGACCA-3’ |
|  | P18: 5’-CCATCGATGAATTCCTGTGCGCATCGGA-3’ |
| pOZ | P19: 5’-GAAGATCTATGACCATGATTACGGATTCACT-3’ |
|  | P20: 5’-GAATGTACATTATTTTTGACACCAGACCA-3’ |
|  | P21: 5’-GAATGTACATAACTGCCTGCGTTGAAGATG-3’ |
|  | P22: 5’-GAAGATCTTGCTGGTCTCCAGTGGTGAA-3’ |
| pDBD-Z | P23: 5’-ACCGGTGAAGGCCTGGATGTTCTT-3’ |
|  | P24: 5’-AGATCTTGACCTGCCGGCCT-3’ |
| pJun-P-CAB | P25:5'CTAGCTAGCCGAACCACCGCCGCCATAGTTCATGACCTTCTGCT 3' |
|  | P26: 5'GGGAATTCCATATGTGCGGTGGTAGAATTGC 3' |
|  | P27: 5'TCCCCCCGGGCACTATAGGGCGAATTGGGTAC 3' |
|  | P28: 5'GCTCTAGACAAGTACCTTGCCGACATCTATG 3' |

**Table S2.** Results of Duncan multiple range test

|  | H | B | C | L | I | F | D | G | E | J | K |
| --- | --- | --- | --- | --- | --- | --- | --- | --- | --- | --- | --- |
| A  K  J  E  G  D  F  I  L  C  B | 526.6***  367.9***  496.1***  516.0***  28.84  4.033  4.347  14.65  23.49  22.75  7.387 | 519.2***  360.6***  488.7***  508.6***  21.46  3.353  3.040  7.260  16.10  15.37 | 503.9***  345.2***  473.3***  493.2***  6.090  18.72  18.41  8.107  0.7333 | 503.1***  431.1***  472.6***  489.2***  5.357  19.45  19.14  8.840 | 512.0***  353.3***  481.4***  501.3***  14.20  10.61  10.30 | 522.3***  363.6***  491.7***  511.6***  24.50  0.313 | 522.6***  363.9***  492.0***  512.0***  24.81 | 497.8***  339.1***  467.2***  487.1*** | 10.62  148.0**  19.91 | 30.54  128.1** | 158.7** |

The data present the differential values of β-galactosidase activity in pairwise comparison. Data without asterisk: no significant difference between two groups. Data with two asterisks ^**^ (*p* < 0.01): significant difference between two groups. Data with three asterisks ^***^ (*p* < 0.001): highly significant difference between two groups. *E. coli* XL1-Blue harboring (A) pOZ; (B) pDBD-Z; (C) pDBD-Z and pP-CAB; (D) pDBD-Jun-Z and pFos-P; (E) pDBD-Jun-Z and pFos-P-CAB; (F) pDBD-Jun-Z and pFos; (G) pDBD-Jun-Z and pP-CAB; (H) no plasmid; (I) pDBD-Z and pFos-P-CAB; (J) pDBD-Jun-Z and pJun-P-CAB; (K) pDBD-Jun-Z and pATF2-P-CAB; (L) pDBD-Z and pBHR68.

**Construction of plasmids**

Plasmid pFos-P-CAB was a derivative of pBHR68 that encoded fusion protein prey (Fos)-PhaP and PHB synthesis operon *phaCAB*. The intermediate plasmid pFos-P was generated by removing the *phaCAB* gene and replacing it with *phaP* gene and *fos* gene in pBHR68. Primers P1 and P2 were used to amplify the long fragment lack of the *phaCAB* gene from pBHR68, also, *phaP* gene was amplified by PCR from *R. eutropha* H16 genome with primers P3 and P4 and then the two fragments were ligated to form pBHR68P using *Nde*I and *Bgl*II sites by DNA ligation kit ver. 2.0 (Takara, Japan). The *fos-linker* gene synthesized by Invitrogen was inserted into pBHR68P between the *Nde*I and *Aat*II sites to form plasmid pFos-P (Fig. S1).

Subsequently, *phaCAB* operon was amplified with primers P5 and P6 and ligated into plasmid pFos-P using *Xma*I and *Xba*I sites, giving rise to pP-Fos-CAB (Fig. S2). The fragment deleting *fos-linker* gene was amplified with primers P7 and P8 from plasmid pP-Fos-CAB, and the fragment was phosphorylated on the 5’ end using T4 polynucleotide kinase (Fermentas, MBI), finally, plasmid pP- CAB was generated by self-ligation (Fig. S2). In addition, the processes of plasmids pP-Jun-CAB and pP-ATF2-CAB reconstruction were similar to plasmid pP-Fos-CAB (figures not shown).

PHB synthesis operon *phaCAB* in pBHR68 was substituted for *fos-linker* to construct pFos (Fig. S3). To do this, primers P9 and P10 were used to amplify *fos-linker,* primers P11 and P12 were used to amplify the fragment cutting off *phaCAB* gene from pBHR68.The two fragments above was ligated by *Nde*I and *Bgl*II sites.

DBD segment of PhaR amplified by PCR with primers P13 and P14 from *R. eutropha* H16 genome was used to replace *phaCAB* in the pBHR68, resulting in pDBD. For amplification of the fragment pBHR68 lacking *phaCAB*, primers P15 and P16 were used. In this construct, restriction sites *Nde*I and *Bgl*II were separately introduced to the two ends of the fragments (Fig. S4). Ultimately, gene *linker-jun* was inserted into pDBD between *Age*I and *Bgl*II sites to obtain pDBD-Jun (Fig. S4).

The *phaP* operon was amplified from *R. eutropha* H16 genome using primers P17 and P18. A restriction site *Cla*I was introduced to the two ends of the *phaP* operon. Subsequently, the fragment digested with *Cla*I was inserted into plasmid pACYC184 in the same restriction site to form pACYC184-OP. When *phaP* was replaced by *lacZ*, pOZ was obtained (Fig. S5). During the plasmid construction, primers P19 and P20 were used to amplify *lacZ*, while primers P21 and P22 were employed to clone the fragment with a deletion of *phaP* in pACYC184-OP (Fig. S5).

pDBD-Jun was digested by *BamH*I and *Hind*III, resulting in a fragment containing *DBD-linker-jun*. This fragment was then inserted into pOZ to form pDBD-Jun-Z (Fig. S6). Deletion of *linker-jun* from pDBD-Jun-Z gave rise to pDBD-Z. During the plasmid construction, primers P23 and P24 were used and this fragment was then phosphorylated on the 5’ end using T4 polynucleotide kinase (Fermentas, MBI), the self-ligation resulted in pDBD-Z (Fig. S6).


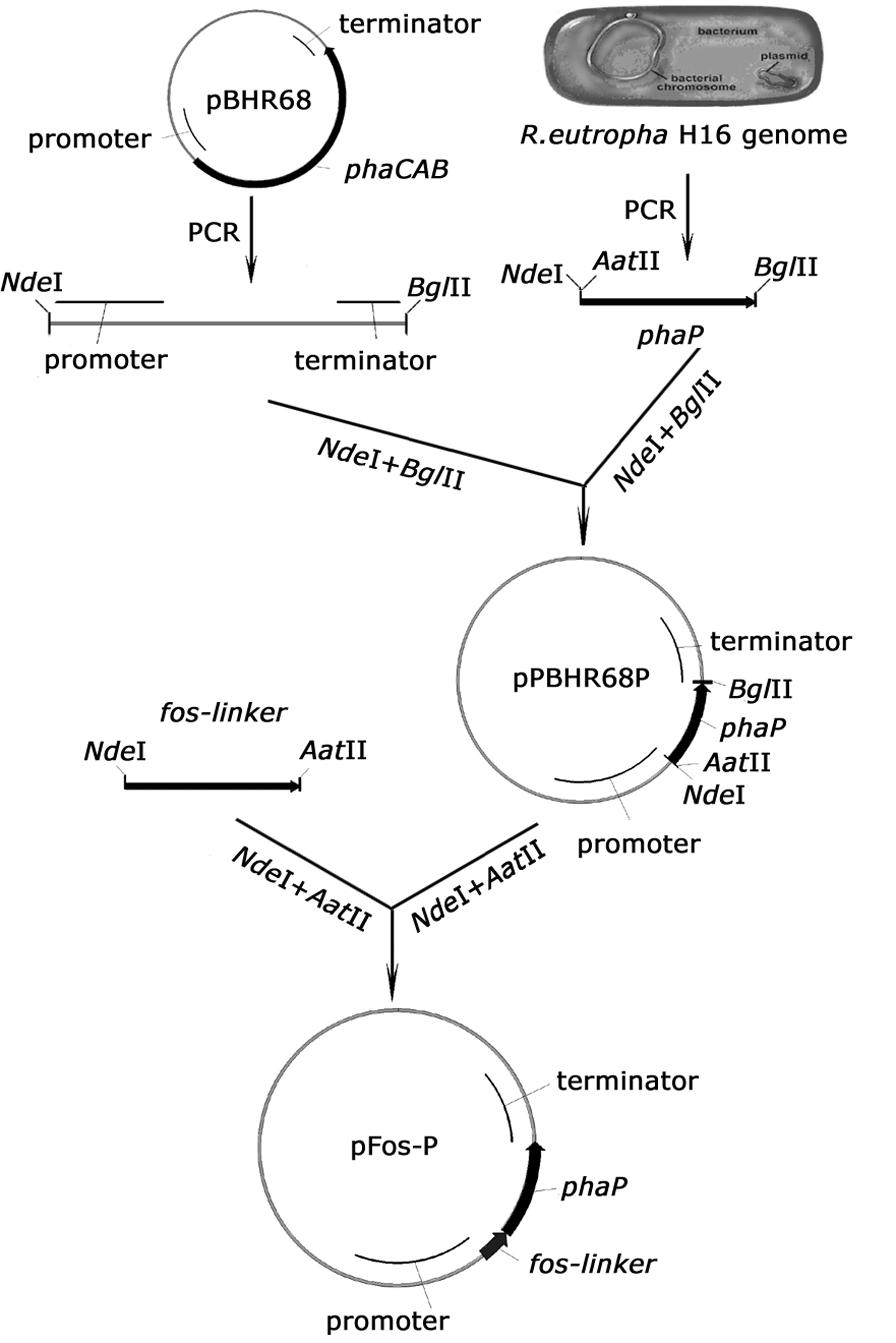


**Figure S1.** Construction of plasmid pFos-P.


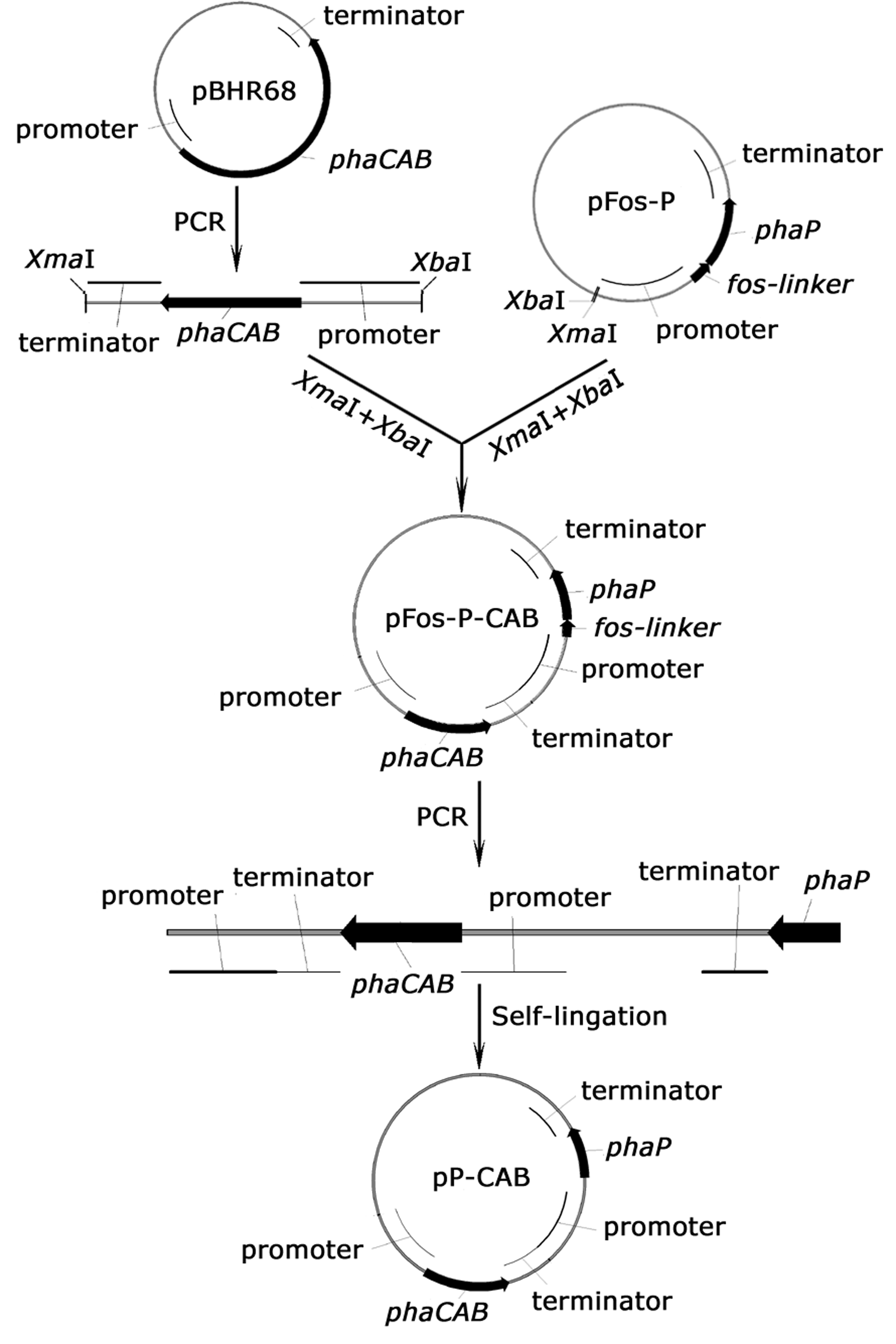


**Figure S2.** Construction of plasmids pFos-P-CAB and pP-CAB.


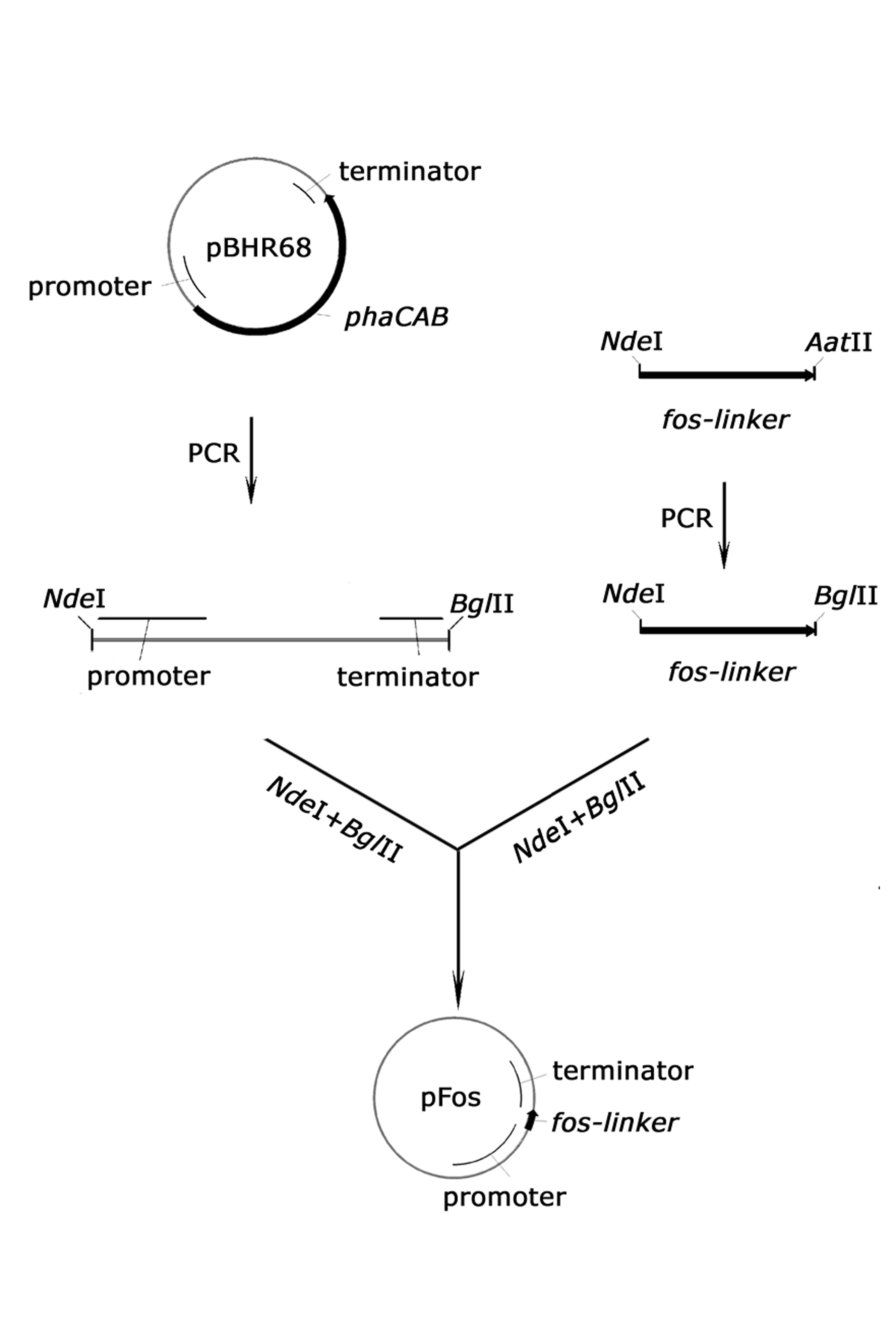


**Figure S3.** Construction of plasmid pFos.


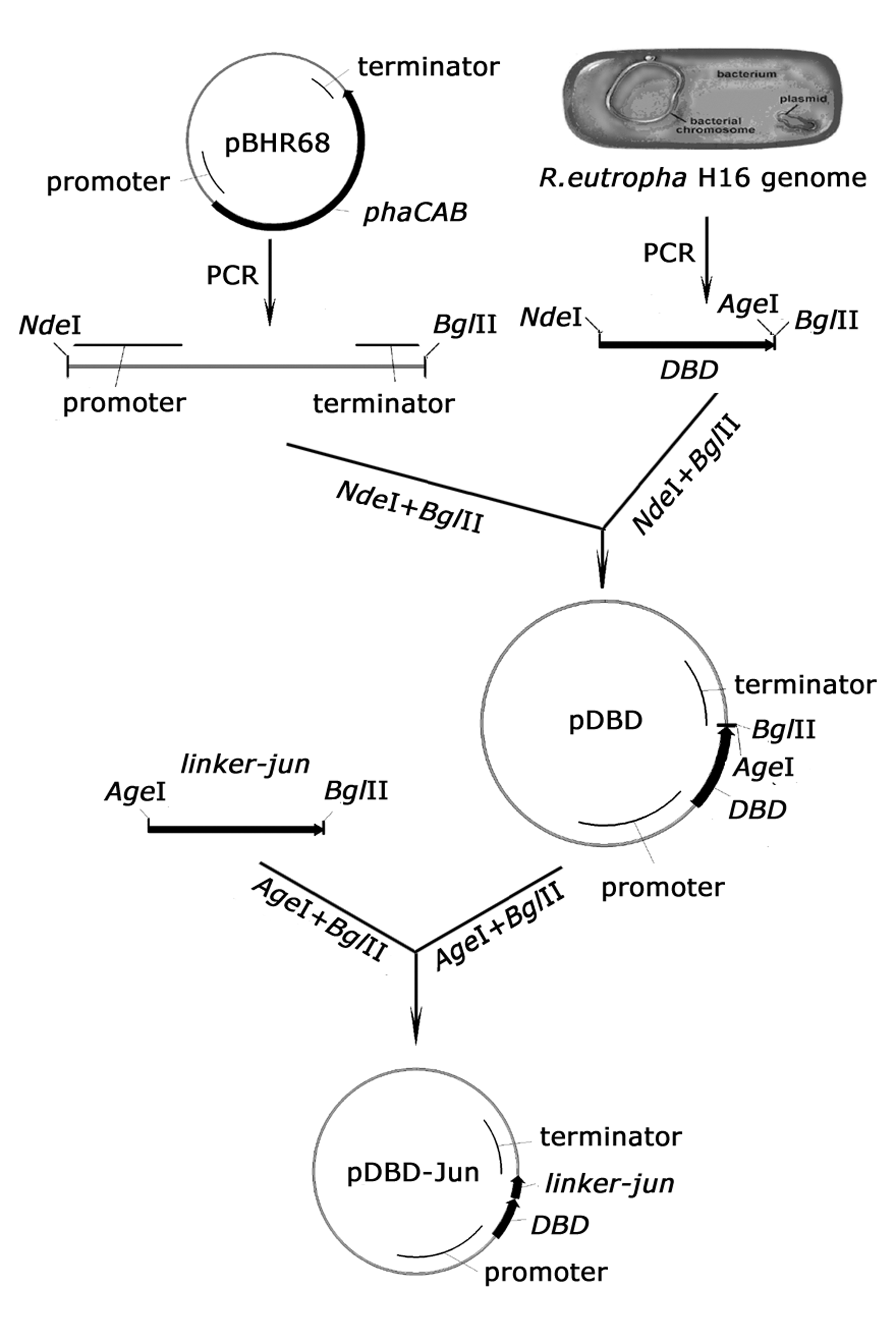


**Figure S4.** Construction of plasmid pDBD-Jun.


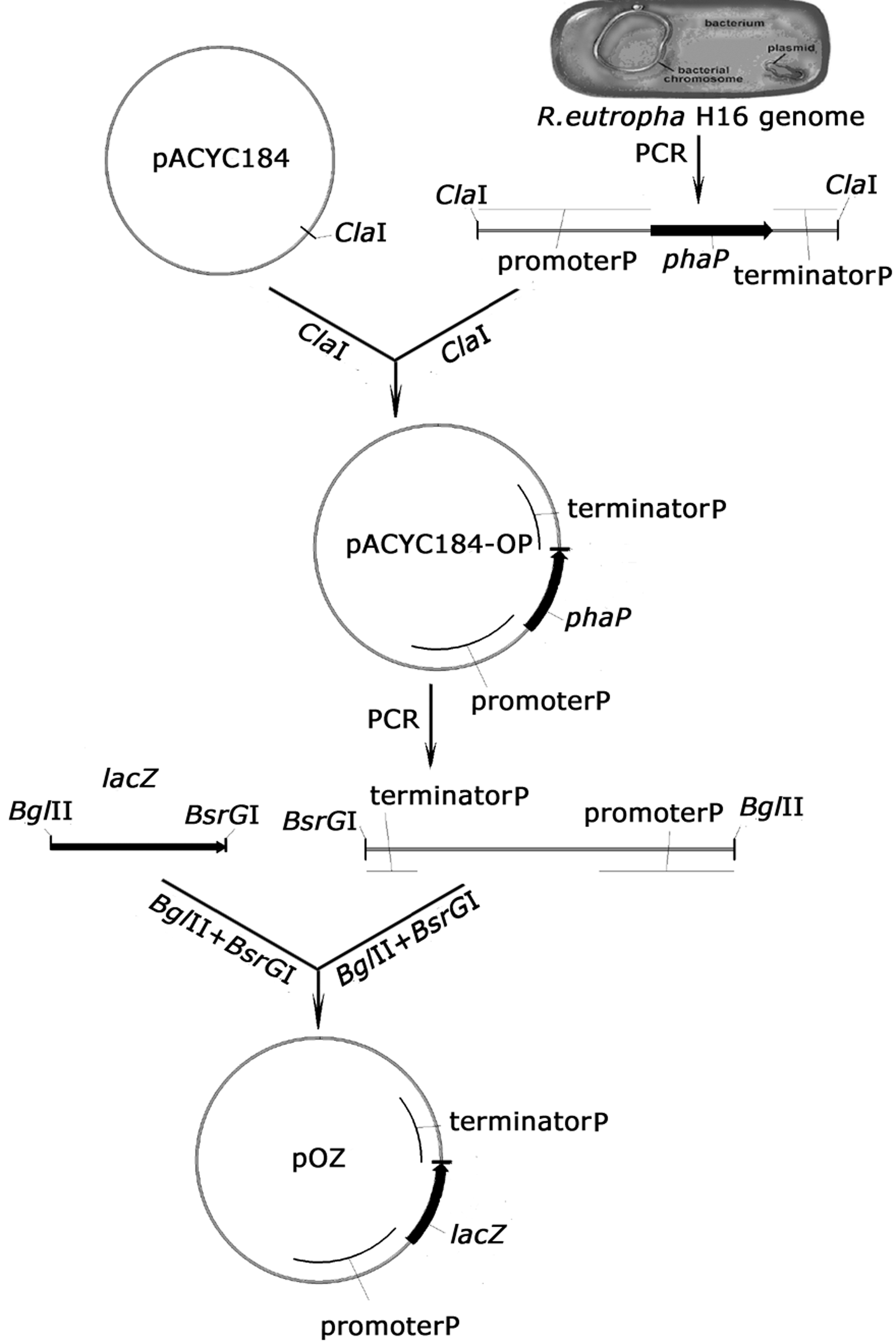


**Figure S5.** Construction of plasmid pOZ.


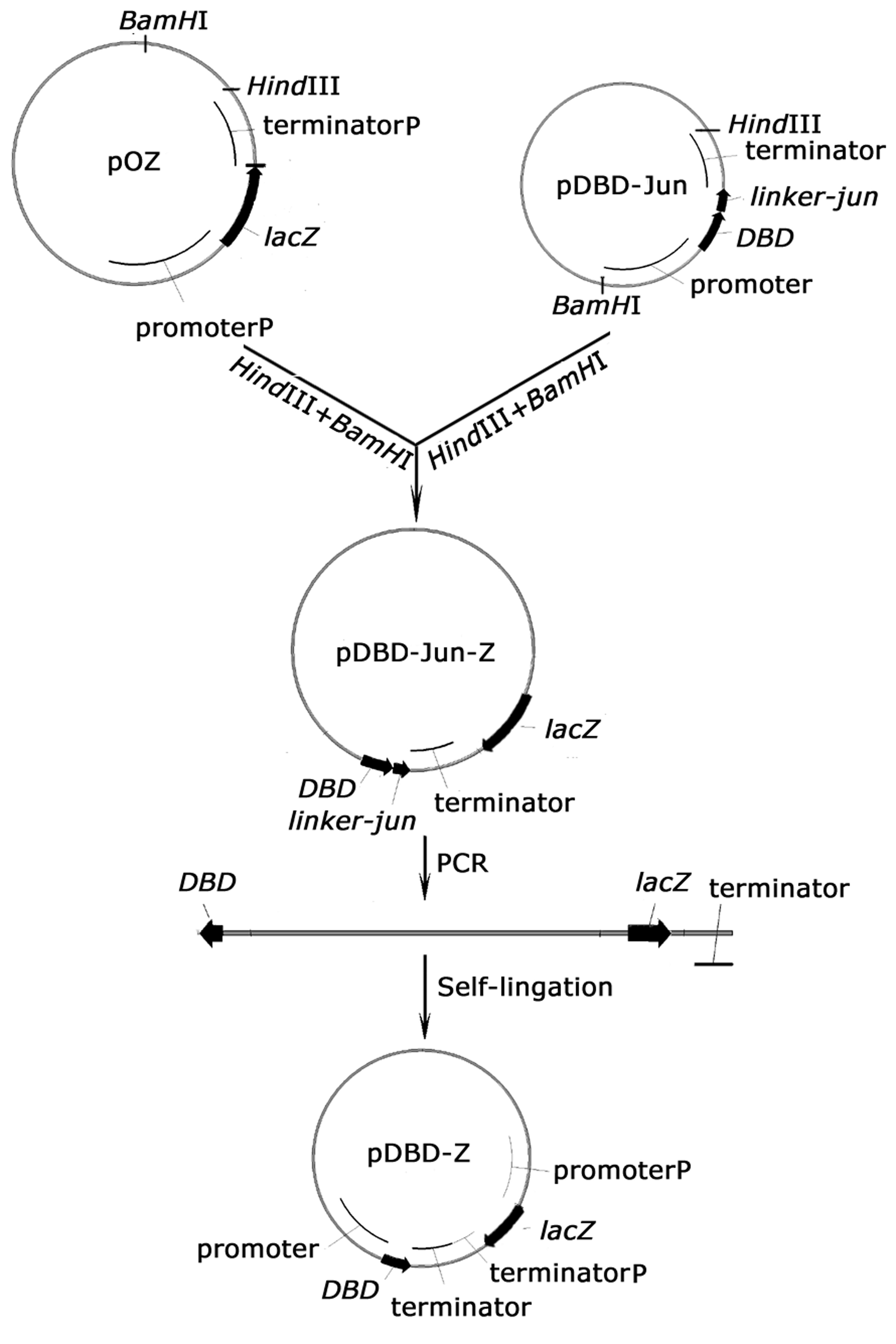


**Figure S6.** Construction of plasmids pDBD-Jun-Z and pDBD-Z.
